# Risk of dementia in patients with atrial fibrillation: Short versus long follow‐up. A systematic review and meta‐analysis

**DOI:** 10.1002/gps.5582

**Published:** 2021-05-27

**Authors:** Marco Zuin, Loris Roncon, Angelina Passaro, Cristina Bosi, Carlo Cervellati, Giovanni Zuliani

**Affiliations:** ^1^ Department of Translational Medicine University of Ferrara Ferrara Italy; ^2^ Department of Cardiology Santa Maria Delle Misericordia Hospital Rovigo Italy

**Keywords:** atrial fibrillation, dementia, elderly, meta‐analysis, systematic review

## Abstract

**Background:**

No previous meta‐analyses have compared the risk of dementia, due to an underlying atrial fibrillation (AF), in the short‐term versus the long‐term period.

**Aim:**

To perform an update meta‐analysis of studies examining the association between AF and dementia and the relative impact of follow‐up period.

**Methods:**

Data were obtained searching MEDLINE and Scopus for all investigations published between 1 January 2000 and March 1, 2021 reporting the risk of dementia in AF patients. The following MeSH terms were used for the search: “Atrial Fibrillation” AND “Dementia” OR “Alzheimer’s disease”. From each study, the adjusted hazard ratio (aHR) with the related 95% confidence interval (CI) was pooled using a random effect model.

**Results:**

The analysis was carried out on 18 studies involving 3.559.349 subjects, of which 902.741 (25.3%) developed dementia during follow‐up. A random effect model revealed an aHR of 1.40 (95% CI: 1.27–1.54, *p* < 0.0001; I^2^ = 93.5%) for dementia in subjects with AF. Stratifying the studies according to follow‐up duration, those having a follow‐up ≥10 years showed an aHR for dementia of 1.37 (95% CI: 1.21–1.55, *p* < 0.0001, I^2^ = 96.6%), while those with a follow‐up duration <10 years has a slightly higher aHR for dementia (HR: 1.59, 95%CI: 1.51–1.67, *p* < 0.0001, I^2^ = 49%). Nine studies showed that the aHR for Alzheimer’s disease (AD) in AF patients was 1.30 (95%CI: 1.12–1.51, *p* < 0.0001, I^2^ = 87.6%).

**Conclusions:**

Evidence suggests that patients with AF have an increased risk of developing dementia and AD. The risk of dementia was slightly higher when the follow‐up was shorter than 10 years.

## Key Points

- The presence of atrial fibrillation increased the risk of developing dementia by 40%
- The presence of atrial fibrillation increased the risk of developing Alzheimer’s disease by 40%
- The risk of developing dementia in patients with atrial fibrillation appears to be inversely related to the length of follow‐up

## 1 INTRODUCTION

Atrial fibrillation (AF) represents the most common cardiac arrhythmia affecting elderly patients,^1^, ^2^ affecting about 9% of adults aged 80 years or older.^3^ In Europe alone, prevalence of AF in 2010 was around 9 million among individuals older than 55 years and is expected to reach 14 million by 2060.^4^

Aging itself exerts significant structural changes on the atrial bundles, characterized by an excessive accumulation of fibrillary collagen in the extracellular space which leads to a progressive age‐dependent cardiomyocyte loss and concomitant fibrosis replacement.^5^ AF is associated with multiple comorbidities, including the development of vascular dementia, but also of the major cause of dementia syndrome, Alzheimer’s disease (AD).^6^, ^7^, ^8^ This association appears to be multifactorial, and no one model will explain the association completely. Cerebrovascular events, such as stroke/transient ischemic attack, but also subclinical abnormalities, in primis microbleeds and chronic cerebral hypoperfusion (CCH), may reasonably account for this observed relationship.^6^, ^9^, ^10^ Consistently, animal studies suggest that long‐term AF decreases cardiac output and may precede CCH and the consequent hypoxia.^6^ In turn, these adverse events impair the clearance and enhance the accumulation of amyloid‐*β* peptides collection in cerebral vessels, therefore increasing AD risk.^10^

A further support on the link between AF and dementia emerged from observational studies showing that, among subjects with either prevalent or incident AF, the treatment with anticoagulant drugs was associated with a decreased risk of cognitive impairment or dementia.^11^, ^12^ However, before speculating about the potential treatment strategies able to reduce the risk of dementia due to an underlying AF, a more precise estimation of that risk, especially in those patients having such arrhythmic disease for a long time, remains essential. Moreover, AF is a progressive disease that becomes more difficult to treat with increasing duration and in this regard, aging plays a fundamental role, also the onset on multiple comorbidities which may tigger and maintain AF.^13^ Compellingly, no previous analyses have compared the risk of dementia, due to an underlying AF, in the short‐term versus the long‐term period. Therefore, in the present manuscript we performed a systematic review and meta‐analysis aimed to evaluate the long‐term relationship (>10 years) between AF and dementia in population‐based studies.

## 2 MATERIALS AND METHODS

The study was performed in accordance with the preferred reporting items for systematic reviews and meta‐analyses (PRISMA) guidelines (Table S1).^14^

### 2.1 Data searching and studies selection

Data were obtained searching MEDLINE and Scopus for all investigations published in English language between 1 January 2000 and 1 March 2021 reporting the risk of dementia in AF patients. The selection of studies was independently conducted by 2 authors (MZ, GZ.) in a blinded fashion. Any discrepancies in study selection were resolved consulting a third author (CC). The following MeSH terms were used for the search: “Atrial Fibrillation” AND “Dementia” OR “Alzheimer’s disease.” Moreover, to ensure comprehensiveness, reference lists of retrieved studies and previous review articles were screened for additional relevant studies. Studies were included if: (1) they provided data regarding the risk of dementia in patients with confirmed AF; (2) the risk of dementia was expressed as adjusted hazard ratio (aHR) with relative 95% confidence (3) they reported information regarding the dementia diagnosis. Conversely, studies reporting the occurrence of AF in patients with mild cognitive impairment (MCI) as well as randomized controlled trials, case reports, review articles, abstracts, editorials/letters, and case series with less than 15 participants were excluded from the analysis. Pre‐clinical studies (i.e., in‐vitro or animal studies) were also excluded by the final analysis. If a study involved the same population, only the most recent investigation was included (overlapping cohort).

### 2.2 Outcomes and data extraction

The primary outcome of the meta‐analysis was the development of any kind of dementia in AF patients. The secondary outcomes were the comparison of dementia risk after considering the duration of the follow‐up (<10 vs. ≥ 10 years) and the correlation between AF and risk of Alzheimer’s disease (AD). For all investigations reviewed we extracted the year of publication, gender (males %), follow‐up duration, total number of participants and dementia patients and methods used for both AF and dementia diagnosis. Two authors (A.P. and C.B.) revised and extracted the data; in case of discrepancies a third author was consulted (C.C.).

### 2.3 Quality of studies

The quality of included studies was graded using the Newcastle‐Ottawa quality assessment scale (NOS).^15^ Specifically, three authors (M.Z., G.Z. and C.C.) performed the quality assessment; in case of discrepancies, a fourth author was consulted and then, the debate was resolved by consensus.

### 2.4 Statistical analysis

From each study, the adjusted hazard ratio (aHR) with the related 95% confidence interval (CI) was pooled using a random‐model while a forest plot was adopted to visually evaluate the results. Statistical heterogeneity between groups was measured using the Higgins I^2^ statistic. Specifically, a I^2^ = 0 indicated no heterogeneity while we considered low, moderate, and high degrees of heterogeneity based on the values of I2 as <25%, 25%–75% and above 75% respectively. Moreover, tau‐squared (τ^2^) was also calculated to see the extent of variation among the effects observed in different studies. To evaluate the publication bias, both the visual inspection of the funnel plots and the Egger’s test were used. A predefined sensitivity analysis (leave‐one‐out analysis) was performed removing one study at the time. To further appraise the impact of potential baseline confounders, meta‐regression analyzed using the length of follow‐up of each study and the latitude of the population enrolled, as moderator variables were performed. Analyses were carried out using comprehensive meta‐analysis software (CMA), version 3. The HRs were compared by using the software R (R software—version 3.6.3) (http://www.r‐project.org/). A *p*‐value <0.05 was considered statistically significant.

## 3 RESULTS

### 3.1 Search results and study characteristics

**FIGURE 1:**
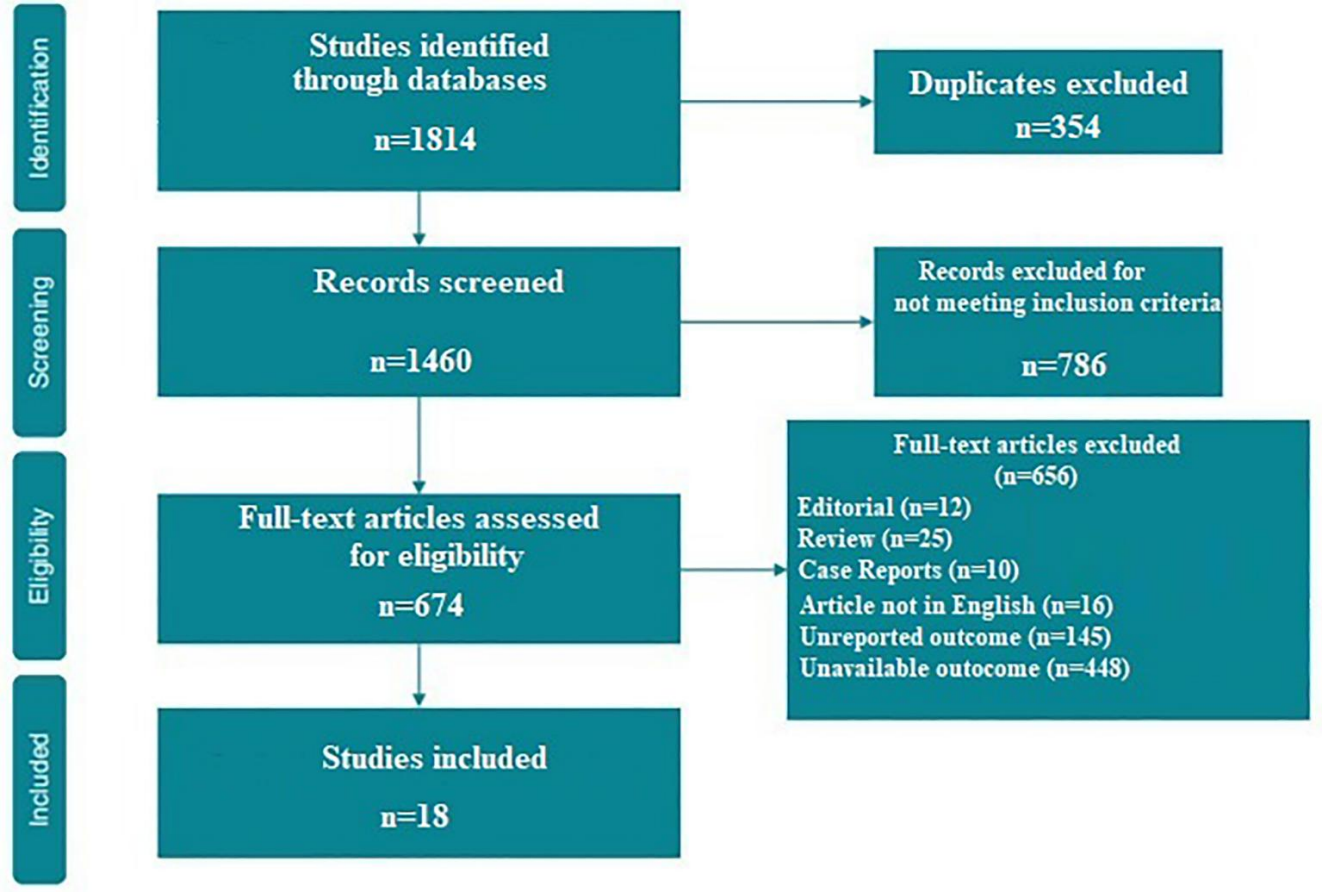
Preferred reporting items for systematic reviews and meta‐analyses (PRISMA) flow chart

A total of 1,460 articles were retrieved after excluding duplicates. The initial screening excluded 425 articles because they did not meet the inclusion criteria, leaving 674 articles to assess for eligibility. Subsequently, after evaluation of the full‐text articles, 656 were excluded and 18 investigations met the inclusion criteria (Figure 1).^11^, ^16^, ^17^, ^18^, ^19^, ^20^, ^21^, ^22^, ^23^, ^24^, ^25^, ^26^, ^27^, ^28^, ^29^, ^30^, ^31^ As shown in Table S2, the number of subjects under oral anti‐coagulant therapy was precisely reported in some of the studies considered in the analysis.^16^, ^19^, ^25^, ^30^, ^32^ Among the 3.559.349 patients enrolled in the reviewed studies, 902.741 (25.3%) developed dementia during follow‐up. The demographical characteristics as well as the criteria adopted to define AF and to diagnose dementia are shown in Table 1.

**TABLE 1:** General characteristics of the studies included into the analysis

Author	Year	Country	Type of study	Design of the study	Total N. of Patients	Age Years (SD)	Patients who developed dementia n (%)	Male %	Inclusion criteria	AF Diagnosis	Dementia Diagnosis	Follow‐up Years and starting year	NOS
Kim et al.	2020	South Korea	National Health insurance cohort	Longitudinal	428,262	N/A	10052 (2%,4%)	54	40‐79 years	ICD codes	ICD codes	9	7
Nah et al.	2020	South Korea	National health insurance cohort	Retrospective	440,826	73 (6)	9086 (2%)	40	≥65 years	ICD codes	ICD codes	10	7
Kim et al.	2019	South Korea	National health insurance cohort	Longitudinal	262,611	71 (5)	38844 (15%)	NR	≥60 years	ICD codes	Korean Dementia screening questionnaire; ICD‐10 codes	8	9
Krawczyk et al.	2019	Canada	Cohort	Retrospective	9791	78 (7)	2460 (25%)	49	≥65 years ‐After ischemic stroke	Medical records; ECG‐H	Medical records	5.5	9
Ding et al.	2018	Sweden	Population‐based	Longitudinal	2685	73 (10)	399 (15%)	37	≥60 years	ECG; ICD codes	DSM‐IV; MMSE; NINDS‐AIREN; NINCDS‐ARDRA	9	8
Chen et al.	2018	US	Community‐based	Longitudinal	12,515	57 (6)	1157 (9%)	44	45‐64 years‐	ICD codes and EEG	MMSE; ICD codes; ARIC‐NCS; DSST	10.6	9
Singh‐Manoux et al.	2017	Great Britain	Cohort	Longitudinal	10,308	NR	912 (9%)	84	45‐69 years	NR	ECG; ICD codes	26.6	9
Marzona et al.	2016	Italy	Population‐based	Retrospective	1,627,631	NR	134837 (8%)	48	≥65 with AF	ICD codes	ICD codes	10	9
De Bruijn et al.	2015	Netherlands	Population‐based	Longitudinal	6194	NR	994 (16%)	40	≥55 years	NR	MMSE, GMSS, DSM‐III	20	8
Liao et al.	2015	Taiwan	National health insurance database	Retrospective	665,330	70 (13)	56901 (8%,6%)	56	≥20 years	NR	ICD codes	15	8
Rusanen et al.	2014	Finland	Population‐based	Longitudinal	1510	50 (6)	127 (8%,4%)	37	No inclusion criteria	ICD codes	DSM‐IV; MMSE	7.8	9
Haring et al.	2013	US	Community‐based	Longitudinal	7479	NR	186 (2%,5%)	0	Postmenopausal women ≥65 years	Self‐report questionnaire	DSM ‐IV; MMSE	8.6	8
Marzona et al.	2012	Several countries	Cohort	Longitudinal	31,506	66 (7)	2157 (7%)	70.2	≥55 years ‐History of CVD or diabetes with evidence of end organ damage^a^	Interview	MMSE	12	8
Dublin et al.	2011	US	Population‐based	Longitudinal	3045	74 (70‐79)	572 (19%)	40	≥65 years	ICD codes	MMSE	6.8	7
Bunch et al.	2010	US	Cohort	Longitudinal	37,025	NR	1535 (4%)	60	No inclusion criteria	ICD codes	DSM‐IV	5	7
Marengoni et al.	2011	Sweden	Population‐based	Longitudinal	685	NR	170 (25%)	NR	≥75 years	ICD codes; medical reports; H&P	DSM‐III R	6	6
Rastas et al.	2007	Finland	Population‐based	Longitudinal	1106	NR	339 (31%)	20	≥85 years	Medical records; ECG; ECG‐H	MMSE; DSM‐III	3.5	69?
Forti et al.	2006	Italy	Cohort	Longitudinal	431	NR	36 (8%,4%)	37	>60 years ‐With mild cognitiveimpairment or normal cognition	H&P	MMSE	4	6

Abbreviations: CDR, clinical dementia rating; ECG, electrocardiography; DSM, diagnostic and statistical manual of mental disorders; GMSS, geriatric mental state schedule; H&P, history and physical examination; ICD, international classification of diseases; MMSE, mini‐mental state examination; NINDS‐AIREN, national institute of neurological disorders and stroke and association international pour la Recherche et l’Enseignement en neurosciences; NINCDS‐ADRDA, national institute of neurological and communicative disorders and stroke and the Alzheimer’s disease and related disorders association; NOS, Newcastle‐Ottawa quality assessment scale; NR, not reported.

^a^ former participants of the study.

The studies included into the meta‐analysis resulted of moderate‐high quality according to the NOS (Table 2).^15^

**TABLE 2:** Quality of the included studies assessed using the Newcastle‐Ottawa quality assessment scale (NOS)

Author	Year	NOS			Total
		Selection	Comparability	Outcome	
Kim et al.	2020	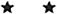	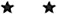	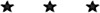	7
Nah et al.	2020	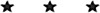	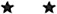	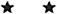	7
Kim et al.	2019	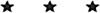	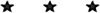	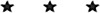	9
Krawczyk et al.	2019	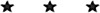	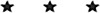	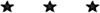	9
Ding et al.	2018	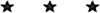	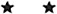	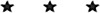	8
Chen et al.	2018	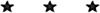	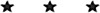	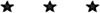	9
Singh‐Manoux et al.	2017	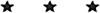	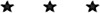	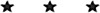	9
Marzona et al.	2016	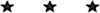	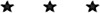	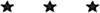	9
De Bruijn et al.	2015	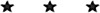	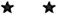	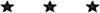	8
Liao et al.	2015	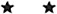	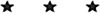	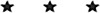	8
Rusanen et al.	2014	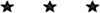	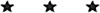	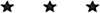	9
Haring et al.	2013	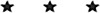	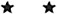	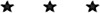	8
Marzona et al.	2012	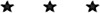	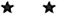	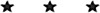	8
Dublin et al.	2011	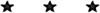	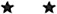	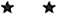	7
Marengoni et al.	2011	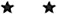	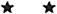	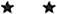	6
Bunch et al.	2010	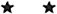	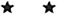	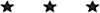	7
Rastas et al.	2007	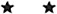	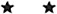	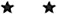	6
Forti et al.	2006	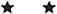	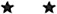	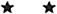	6

### 3.2 Atrial fibrillation and dementia risk

**FIGURE 2:**
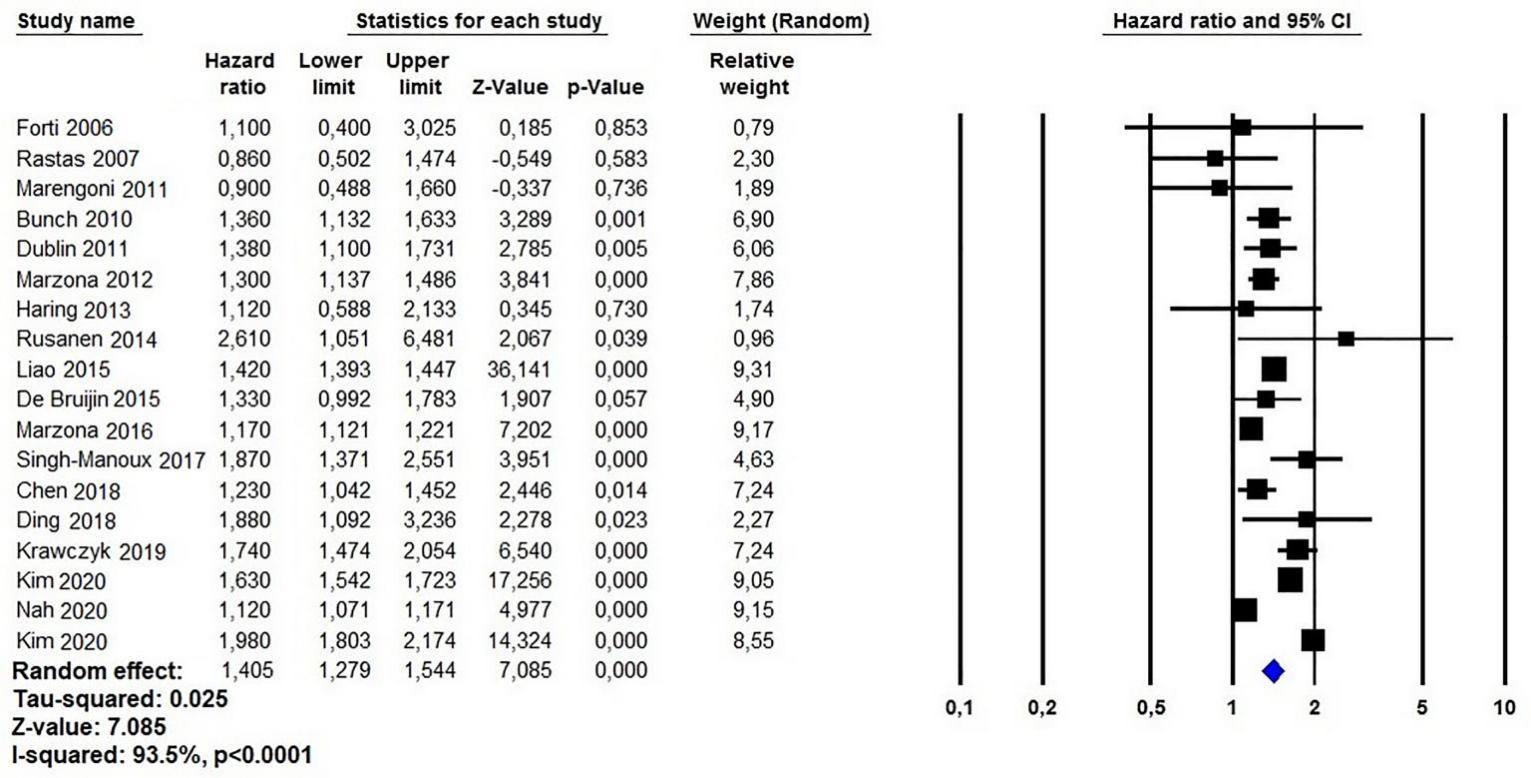
Forest plots investigating the overall risk of dementia in atrial fibrillation patients

**FIGURE 3:**
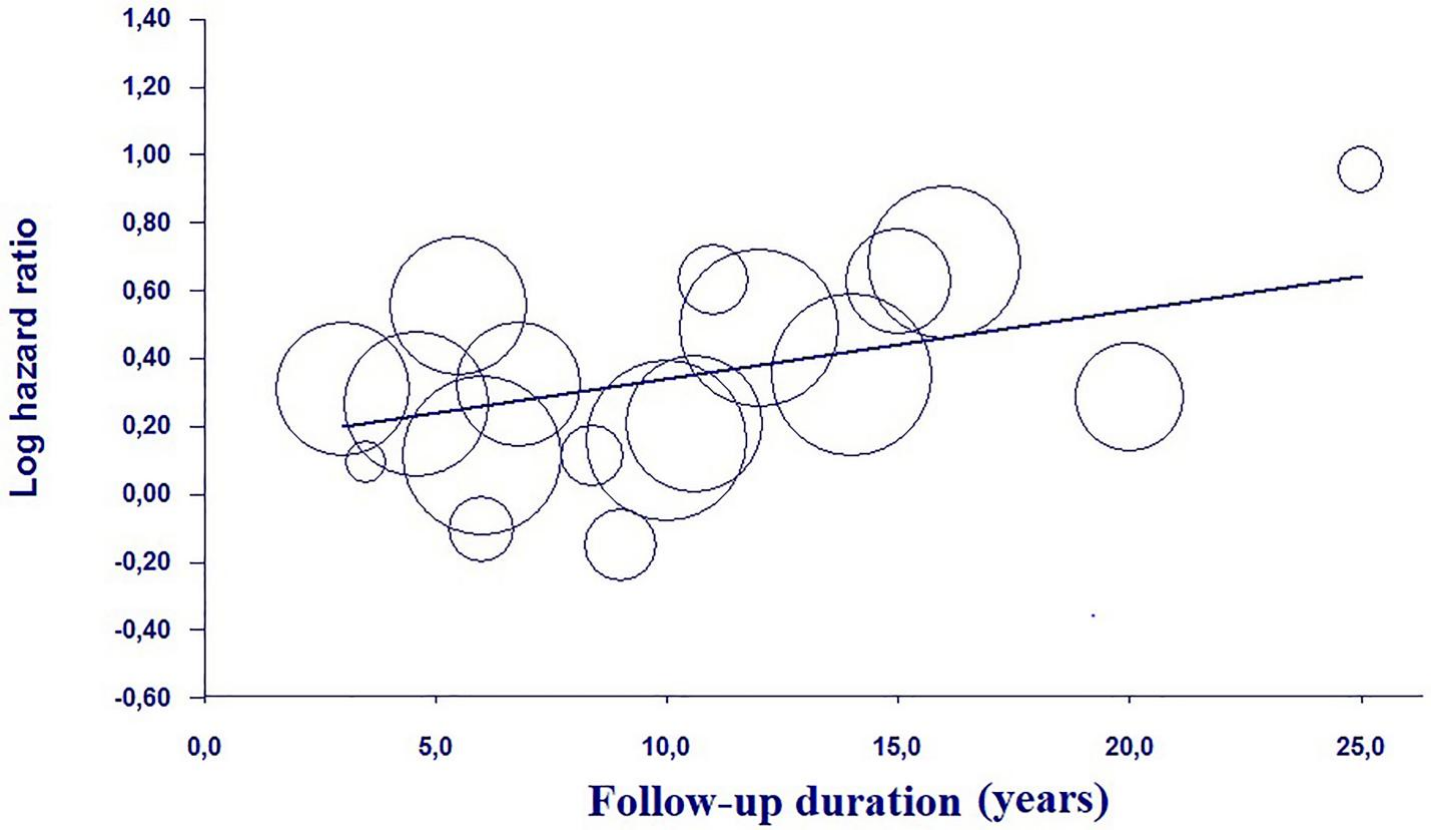
Meta‐regression analysis examining the risk of dementia, due to an underlying atrial fibrillation, respect to the follow‐up length of revised studies

All 18 studies evaluated the relationship between AF and dementia risk. The variables used by each study reviewed for adjustments are presented in Table 3. The pooled analysis, using a random effect model revealed an aHR of 1.40 (95%CI: 1.27–1.54, *p* < 0.0001; I^2^ = 93.5%) for dementia in AF subjects (Figure 2). Both the funnel plot (Figure S1) and Egger’s test (*t* = 0.106, *p* = 0.916) revealed absence of bias. One‐by‐one exclusion of the studies from the analysis slightly changed the combined HR, which remained statistically significant across a range from 1.42 (95% CI: 1.29–1.56, *p* < 0.0001) to 1.39 (95% CI: 1.27–1.53, *p* < 0.0001), suggesting that no single study had an undue impact on the combined HR. Meta‐regression analysis, using the length of follow‐up period of each study reviewed, showed a significant direct relationship with the risk of dementia due to AF (Coeff. 0.020, 95% CI: 0.002–0.039, *p* = 0.02; Figure 3). Conversely, no interaction was present using the latitude of the population enrolled as moderator variables (Coeff. 0.003, 95% CI: −0.005–0.012, *p* = 0.40).

**TABLE 3:** Confounding variables used for the COX‐regression adjustment in each study reviewed

Author	Year	Age	Gender	Education	MMSE	SBP	DBP	BMI	Serum Folate	HT	HF	DM	AMI	Dys	CKD	TIA	Stroke	CHD	MCI	APOE	Drugs	Other
Kim et al.	2020	X	X	‐	‐	X	X	‐	‐	X	X	X	X	‐	‐	‐	‐	‐	‐	‐	‐	PAD; osteoporosis; COPD; Chol.; economic status; Smoking; hb; ChA2DS2‐VASc
Nah et al.	2020	X	X	‐	‐	‐	‐	‐	‐	X	X	X						X				CAD
Kim et al.	2019	X	X	‐	‐	X	X	‐	‐	X	X	X		X	‐	‐	‐	‐	‐	‐	X (ACEI; ARBs; CCBS; asa; digoxin; statins)	Serum glucose; Chol; serum creatinine; COPD; alcohol; cognitive function
Krawczyk et al.	2019	X	X	‐	‐	‐	‐	‐	‐	X	X	X		X			X				X (anticoagulants)	CAD, smoking
Ding et al.	2018	X	X	X	‐	‐	‐	X	X	X		X	‐	‐	‐	‐	‐	‐	‐	‐	‐	Smoking; alcohol; physical activity; CAD Chol.
Chen et al.	2018	X	X	X	‐	X	X	X	‐	‐	X	‐	‐	‐	‐	‐	X	‐	‐	X	‐	Race; smoking; CAD;
Singh‐Manoux et al.	2017	X	X	X	‐	‐	‐	‐	‐	X	X	X	‐	‐	‐	‐	‐	‐	‐	‐	X (cardiovascular)	Race; alcohol; smoking; physical activity; CVD
Marzona et al.	2016	X	X		‐	‐	‐	‐	‐	‐	‐	X	X	‐	‐	‐	X	‐	‐	‐	X	PAD hospitalizations
De Bruijn et al.	2015	X	X	X	‐	X	X	X	‐	‐	X	X	‐	‐	‐	‐	‐	X	‐	X	Statins	Smoking; Chol.; C‐HDL; CAD
Liao et al.	2015	X	X		‐	‐	‐	‐	‐	X	X	X	‐	X	X	X	X	‐	‐	‐	Aspirin; Clopidogrel; warfarin; ACEI; statins	COPD; Cancer; CCI
Rusanen et al.	2014	‐	X	X	‐	X		X	‐	‐	‐	X	‐	‐	‐	‐	X	‐	‐	X	‐	Chol.; physical activity
Haring et al.	2013	X	‐	X	X	‐	‐	X	‐	X	‐	X	X	‐	‐	‐	‐	‐	‐	‐	‐	Race: Smoking; alcohol; depression; anthropometric
Marzona et al.	2012	X	X	X	X	X	‐	‐	‐	‐	X	X	X	‐	‐	X	X	‐	‐	‐	BB; stati; ACEI; ARBs; anticoagulants	Albuminuria; serum creatinine
Dublin et al.	2011		X	X	‐	X	X	‐	‐	‐	X	X	‐	‐	‐	‐	X	X	‐	‐	‐	‐
Marengoni et al.	2011	X	X	X	X	‐	‐	‐	‐	X	‐	‐	‐	‐	‐	‐	‐	‐	‐	X	Antithrombotic	‐
Bunch et al.	2010	X	X	‐	‐	‐	‐	‐	‐	‐	X	X	X	X	X	X	X	‐	‐	‐	‐	‐
Rastas et al.	2007	X	X	‐	‐	‐	‐	‐	‐	X	X	X	X	‐	‐	‐	‐	‐	‐	‐	‐	‐
Forti et al.	2006	X	X	X	X	‐	X	X	X	‐	‐	‐	‐	‐	‐	‐	‐	‐	X	‐	‐	‐

Abbreviations: ACEI, ace inhibitors; AMI, acute myocardial infarction; ARBs, angiotensin II receptor blockers; BBs, Beta‐blockers; BMI, body mass index; CAD, coronary artery disease; CCI: Charlson’s comorbidity index; CHD, chronic heart disease; Chol., total serum cholesterol; CKD, chromic kidney disease; COPD, chronic obstructive pulmonary disease; DBP, diastolic blood pressure; DM, diabetes mellitus; Dy, dyslipidaemia; Hb, hemoglobin; HT, arterial hypertension; HF, heart failure; MCI, mild cognitive impairment; MMSE, mini‐mental‐state examination; PAD, peripheral artery disease; SBP, systolic blood pressure; TIA, transient ischemic attack.

### 3.3 Risk of dementia in patients with atrial fibrillation based on follow‐up length

**FIGURE 4:**
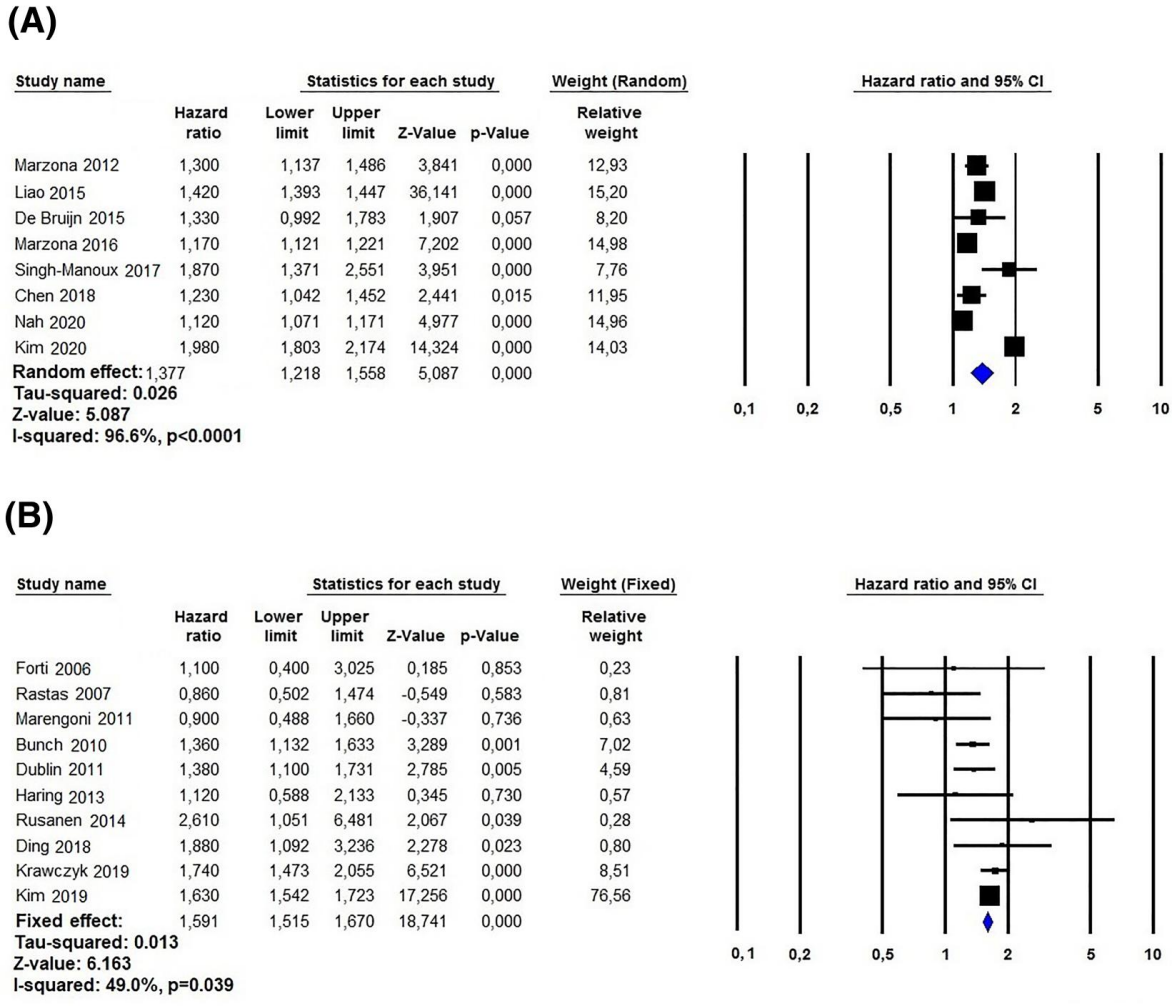
Study with follow‐up ≥10 (A) and <10 years (B) investigating the relationship between atrial fibrillation and the risk of dementia

The studies reviewed were further stratified according to the follow‐up duration to determine the risk of AF over time. Specifically, eight studies, based on 3.232.981 subjects, had a follow‐up ≥10 years [18,21–25,29–30]; in these patients, the aHR for dementia was 1.37 (95%CI: 1.21–1.55, *p* < 0.0001, I^2^ = 96.6%; Figure 4, panel A). On the other hand, the 10 investigations with a follow‐up duration <10 years [13–17,19–20, 26–28], based on 326.386 patients, showed a slightly higher aHR for dementia (HR: 1.59, 95%CI: 1.51‐1.67, *p* < 0.0001, I^2^ = 49%) (Figure 4, panel B). No publication biases were observed at the Egger’s test for the studies with a follow up ≥10 and < 10 years (t = 0.155, *p* = 0.881 and t = 1.706, *p* = 0,126, respectively). The relative funnel plots are showed in Figure S2 (Panels A and B, respectively).

### 3.4 Risk of Alzheimer’s disease in patients with atrial fibrillation

**FIGURE 5:**
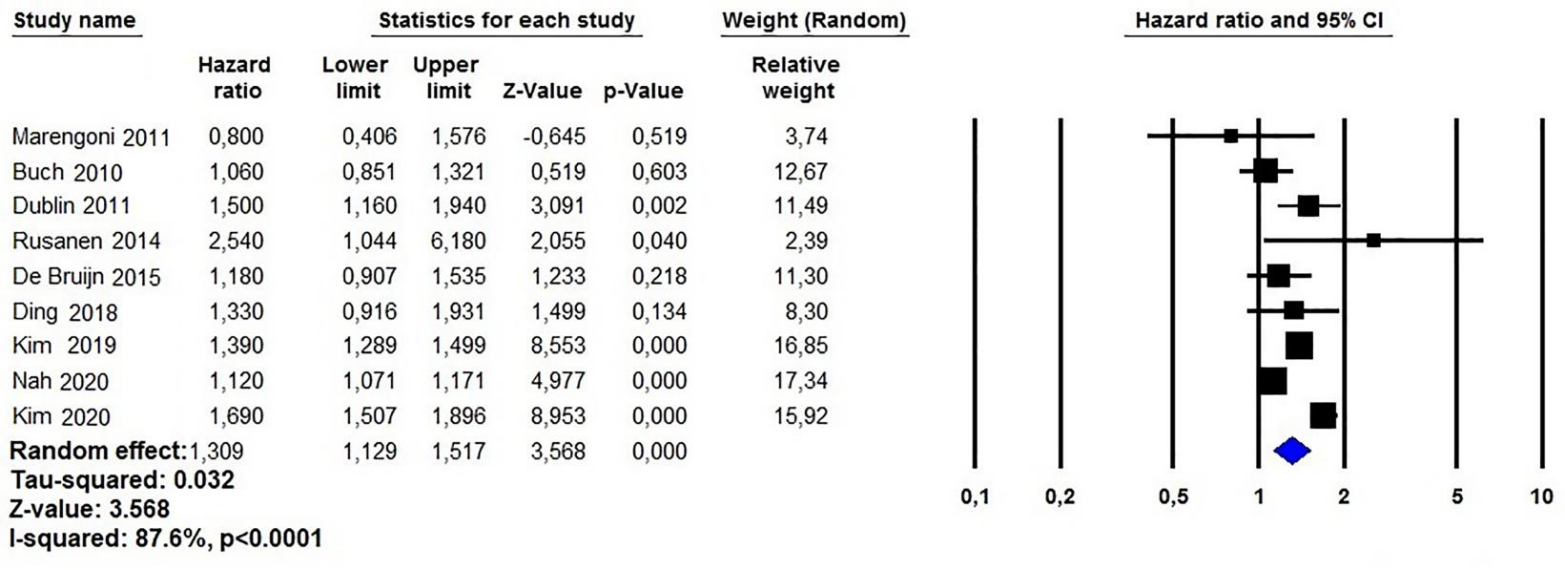
Association between atrial fibrillation and Alzheimer disease risk

Nine investigations performed a sub‐analysis on the risk of developing AD in patients with AF. As showed in Figure 5, the aHR was 1.30 (95%CI: 1.12–1.51, *p* < 0.0001, I^2^ = 87.6%). Again, no bias was detected using the Egger’s test (t = 0.898, *p* = 0.398) or by the visual assessment using the funnel plot (Figure S3).

## 4 DISCUSSION

Our meta‐analysis confirms previous evidence showing an increased risk of dementia in patients affected by AF.^20^, ^21^, ^26^, ^28^ Specifically, an additional 40% risk of developing dementia was observed in patients with AF compared to individuals without this arrythmia. The risk was tendentially higher in the studies with a follow‐up <10 years (+59%), compared to those with a follow‐up length ≥10 years (+37%). Besides the overlap of the confidence intervals of the HR for the studies <10 and ≥10 years, the difference was statistically significant (*p* < 0.02); however, it is well‐known that the effect of a risk factor may vary during follow‐up. Two phenomena may have contributed to the trend we observed: (i) as for other risk factors (e.g. plasma cholesterol, overweight/obesity) it is likely that the deleterious effect of AF may be reduced over the years (ii) since age is the strongest known risk factor for dementia it causes an important increase in the incidence of the disease also in subjects without AF, thus reducing the HR in the long‐term period.

Moreover, patients with AF had an additional 30% risk of developing AD compared to subjects without AF, and this confirms data from literature showing that AF is not only a risk factor for the form of dementia more directly linked with vascular dysfunction, that is vascular dementia (VD).^10^, ^19^, ^33^ Indeed, different pathophysiological mechanisms have been related to the increased risk of dementia in AF patients, including chronic brain hypoperfusion, silent cerebral infarctions, sub‐cortical white matter lesions, systemic and cerebral chronic inflammation^33^, ^34^, ^35^, ^36^; interestingly, all these conditions have been associated with both AD and VD.^35^

The present results highlight that follow‐up period plays a pivotal role on the risk of dementia in AF patients. Indeed, recent investigations have elucidated the progressive nature of AF, that becomes more difficult to treat with increasing duration. This aspect is believed to be mainly due to the occurrence of electrical, contractile, and structural remodeling of the atria, which creates a fertile environment for the propagation of AF.^37^, ^38^, ^39^ Furthermore, AF generally occurs in the setting of underlying heart disease, such as coronary artery disease, hypertension, valve disease, congestive heart failure, and thyroid dysfunction, which may both trigger and maintain the arrhythmia. Moreover, the occurrence of such comorbidities naturally increases with aging,^40^, ^41^ therefore, a longer follow‐up may be more adequate to discriminate the impact of a progressive disease on these patients. Despite sensitivity analysis excluded that no single study had an undue impact on the combined HR, meta‐regression analysis showed that the length of follow‐up duration was a significant source of statistical heterogeneity. Conversely, the latitude of the populations enrolled did not explained the heterogeneity, potentially suggesting that life quality and/or the standard of care did not act as confounding factors.

Among the potential strategies suggested for the reduction of dementia onset due to an underlying AF, some investigations have suggested the potential benefices obtained from the administration of oral anticoagulants.^12^, ^42^ Unfortunately, these drugs remain under administered in elderly patients with dementia, even in the presence of AF.^43^, ^44^ Other findings showed that more invasive treatment, such as catheter ablation, may be another optional treatment to reduce the burden of dementia in these patients, but in most of cases but it remains an invasive procedure with related risk especially in elderly subjects with serval comorbidities and a potential source of silent strokes and cognitive impairment.^23^, ^45^ From a pathophysiological perspective, dementia and AF seems to be linked by the appearance of micro embolic events as well as microinfarcts, as those observed in AD patients.^24^, ^46^ Proinflammatory states are actively implicated in both the genesis and perpetuation of AF, as well as in the promotion of hypercoagulability and thrombus formation, predisposing to stroke.^29^ Previous analyses have demonstrated that the timing for the initiation of oral anticoagulation, and the quality of the drug, had a pivotal role in decreasing the risk of dementia in these patients.^47^, ^48^ Indeed, patients treated with warfarin with a lower TTR, are at higher risk of dementia.^22^ However, some of these issues have been partially overcome with the recent introduction of novel oral anticoagulant agents (NOACS). NOACs, such as dabigatran, rivaroxaban, and apixaban are at least as effective as warfarin in preventing strokes in patients with AF. Moreover, these drugs reduce the risk of intracranial hemorrhage, which represents one of the most important adverse events of oral anticoagulation in elderly with AF.^49^ In this regard, a recent meta‐analysis comparing NOAC and Warfarin has shown that the former significantly reduced the occurrence of dementia and bleeding events.^18^ We cannot assess this aspect in our analysis, since the studies reviewed did not systematically report type, duration, and quality of anticoagulation treatment. Moreover, this aspect was beyond the aim of our study which was to provide an updated evaluation of the risk of developing dementia in AF patients. In a similar fashion, the therapeutic strategy adopted for the treatment of AF in the cohorts included in our analysis could not be evaluated (i.e., Rhythm Control vs. Rate Control). Notably, it has been demonstrated that management of rate and rhythm can reduce the cognitive decline due the generation of a steady and predictable heart rate, in terms of R‐R interval.^1^ Indeed, variance in R‐R intervals coupled lead to loss of atrioventricular synchrony, resulting in reduced cerebral blood flow that causes repetitive hypoperfusions at the arteriolar and hypertensive events at the capillary level.^50^ Also, the use of catheter ablation has been related with a lower risk of dementia and AD but, due the invasive nature of the procedure and the relevant number of comorbidities in elderly patients, a more conservative treatment is generally preferred.^51^

Compared to the latest meta‐analysis available on this topic,^52^ our study added data from 1.144.175 subjects, which represent a significant population, helpful in further elucidating the relationship between AF and dementia. Moreover, this and an earlier meta‐analyze on the same topic comprehensively included patients with dementia and MCI,^52^, ^53^ whereas our investigation specifically focused on dementia. Of interest, also the mentioned studies found a significant association between AF and risk of incidence dementia, with the resulting HRs which was similar to ours: 1.42 (95% CI 1.17–1.72)^52^ and 1.36, (95% CI: 1.23–1.51).

Our analysis evidence how the preservation of cognitive performance and prevention of dementia should be fundamental goals in the management of elderly patients with AF. Besides the traditional cardiovascular evaluation, by using the CHADS2 and CHA2DS2‐VASc, to evaluate the risk of stroke and consequent need of anticoagulation therapy, a multidimensional geriatric evaluation should be recommended as an integral part of the management of these patients when suggestive symptoms for mild cognitive impairment or dementia are detected to limit the AF progression.

The present study suffers from several limitations. Despite the use of a random‐effect model, a high heterogeneity was observed in the overall analysis, as well as after dividing the reviewed studies according to the duration of the follow‐up. Probably, this aspect depends on the inclusion criteria of participants, differences in sample size, methodological quality, demographic and ethno‐racial characteristics of the study populations, various covariate assessments, different length of follow‐up periods, types of dementia, and inherited bias from the original investigations. In particular, the different variables used for the statistical adjustments by each study may have contributed to the observed high heterogeneity. A further important limitation and source of heterogeneity of the meta‐analysis is the lack of information about the number of oral anti‐coagulant users (as well as the type of drugs) of the majority of the examined studies.

## 5 CONCLUSIONS

Patients with AF have an increased risk of developing dementia and AD, and the risk is slightly higher in the studies with a follow‐up shorter than 10 years. Further analyses are needed to confirm our results and to assess the potential benefit of a more aggressive therapy in those patients with AF and long‐life expectances. Indeed, elderly patients generally experience different comorbidities limiting the use of interventional treatments and therefore forcing to a medical treatment, which in several cases is not conclusive or allows some relapse of the arrhythmias with hemodynamic consequences and or complications. Therefore, it appears useful to adopt a patient’s tailored approach also considering the risk of dementia in the long‐term period which should promote the resolution of AF and potentially avoid watchful within approaches also if the arrhythmia is well tolerated and without hemodynamic effects.

## CONFLICTS OF INTEREST

All authors declare that they have no conflicts of interest.

## ETHICAL STATEMENT

Not necessary (systematic review).

## Supporting information

Supplementary Material 1

Supplementary Material 2

Supplementary Material 3

Supplementary Material 4

Supplementary Material 5

## Data Availability

Data sharing is not applicable to this article as no new data were created or analyzed in this study.

## References

[1] Efimova I , Efimova N , Chernov V , Popov S , Lishmanov Y . Ablation and pacing: improving brain perfusion and cognitive function in patients with atrial fibrillation and uncontrolled ventricular rates. Pacing Clin Electrophysiol. 2012;35(3):320‐326. 10.1111/j.1540-8159.2011.03277.x 22126258

[2] Krijthe BP , Kunst A , Benjamin EJ , et al. Projections on the number of individuals with atrial fibrillation in the European Union, from 2000 to 2060. Eur Heart J. 2013;34(35):2746‐2751. 10.1093/eurheartj/eht280 23900699 PMC3858024

[3] Nantsupawat T , Nugent K , Phrommintikul A . Atrial fibrillation in the elderly. Drugs Aging. 2013;30(8):593‐601. 10.1007/s40266-013-0094-8 23709402

[4] Kornej J , Börschel CS , Benjamin EJ , Schnabel RB . Epidemiology of atrial fibrillation in the 21st century. Circ Res. 2020;127(1):4‐20. 10.1161/CIRCRESAHA.120.316340 32716709 PMC7577553

[5] Biernacka A , Frangogiannis NG . Aging and cardiac fibrosis. Aging Dis. 2011;2(2):158‐173. 21837283 PMC3153299

[6] Bunch TJ . Atrial fibrillation and dementia. Circulation. 2020;142(7):618‐620. 10.1161/CIRCULATIONAHA.120.045866 32804567

[7] Pandit SV , Jalife J . Aging and atrial fibrillation research: where we are and where we should go. Heart Rhythm. 2007;4(2):186‐187. 10.1016/j.hrthm.2006.11.011 17275754 PMC1849951

[8] Saglietto A , Matta M , Gaita F , Jacobs V , Bunch TJ , Anselmino M . Stroke‐independent contribution of atrial fibrillation to dementia: a meta‐analysis. Open Heart. 2019;6(1):e000984. 10.1136/openhrt-2018-000984 31217998 PMC6546265

[9] Goos JDC , Teunissen CE , Veerhuis R , et al. Microbleeds relate to altered amyloid‐beta metabolism in Alzheimer's disease. Neurobiol Aging. 2012;33(5):e1–9. 10.1016/j.neurobiolaging.2011.10.026 22118945

[10] Ihara M , Washida K . Linking atrial fibrillation with Alzheimer's disease: epidemiological, pathological, and mechanistic evidence. J Alzheimers Dis. 2018;62(1):61‐72. 10.3233/JAD-170970 29439352 PMC5817903

[11] Ding M , Fratiglioni L , Johnell K , et al. Atrial fibrillation, antithrombotic treatment, and cognitive aging. Neurology. 2018;91(19):e1732‐e1740. 10.1212/WNL.0000000000006456 30305443 PMC6251601

[12] Zeng D , Jiang C , Su C , Tan Y , Wu J . Anticoagulation in atrial fibrillation and cognitive decline. Med Baltim. 2019;98(7):e14499. 10.1097/MD.0000000000014499 PMC6408007 30762777

[13] Van Gelder IC , Hemels MEW . The progressive nature of atrial fibrillation: a rationale for early restoration and maintenance of sinus rhythm. Europace. 2006;8(11):943‐949. 10.1093/europace/eul107 16973685

[14] Liberati A , Altman DG , Tetzlaff J , et al. The PRISMA statement for reporting systematic reviews and meta‐analyses of studies that evaluate health care interventions: explanation and elaboration. PLoS Med. 2009;6(7):e1000100. 10.1371/journal.pmed.1000100 19621070 PMC2707010

[15] Wells GA , Shea B , O’Connell D , et al. The Newcastle‐Ottawa Scale (NOS) for Assessing the Quality of Nonrandomised Studies in Meta‐Analyses. Ottawa ON: Ottawa Hospital Research Institute. Ottawa Ottawa Hosp Res Institute; 2012.

[16] De Bruijn RFAG , Heeringa J , Wolters FJ , et al. Association between atrial fibrillation and dementia in the general population. JAMA Neurol. 2015;72(11):1288. 10.1001/jamaneurol.2015.2161 26389654

[17] Chen LY , Norby FL , Gottesman RF , et al. Association of atrial fibrillation with cognitive decline and dementia over 20 Years: the ARIC‐NCS (atherosclerosis risk in communities neurocognitive study). J Am Heart Assoc. 2018;7(6):e007301. 10.1161/JAHA.117.007301 PMC5907543 29514809

[18] Cheng W , Liu W , Li B , Li D . Relationship of anticoagulant therapy with cognitive impairment among patients with atrial fibrillation: a meta‐analysis and systematic review. J Cardiovasc Pharmacol. 2018;71(6):380‐387. 10.1097/FJC.0000000000000575 29528873

[19] Dublin S , Anderson ML , Haneuse SJ , et al. Atrial fibrillation and risk of dementia: a prospective cohort study. J Am Geriatr Soc. 2011;59(8):1369‐1375. 10.1111/j.1532-5415.2011.03508.x 21806558 PMC3289545

[20] Forti P , Maioli F , Pisacane N , Rietti E , Montesi F , Ravaglia G . Atrial fibrillation and risk of dementia in non‐demented elderly subjects with and without mild cognitive impairment (MCI). Archives Gerontology Geriatrics. 2007;44:155‐165. 10.1016/j.archger.2007.01.023 17317449

[21] Gorelick PB , Scuteri A , Black SE , et al. Vascular contributions to cognitive impairment and dementia. Stroke. 2011;42(9):2672‐2713. 10.1161/STR.0b013e3182299496 21778438 PMC3778669

[22] Jacobs V , Woller SC , Stevens S , et al. Time outside of therapeutic range in atrial fibrillation patients is associated with long‐term risk of dementia. Heart Rhythm. 2014;11(12):2206‐2213. 10.1016/j.hrthm.2014.08.013 25111326

[23] Kim D , Yang P‐S , Sung J‐H , et al. Less dementia after catheter ablation for atrial fibrillation: a nationwide cohort study. Eur Heart J. 2020;41(47):4483‐4493. 10.1093/eurheartj/ehaa726 33022705

[24] Kim D , Yang P‐S , Yu HT , et al. Risk of dementia in stroke‐free patients diagnosed with atrial fibrillation: data from a population‐based cohort. Eur Heart J. 2019;40(28):2313‐2323. 10.1093/eurheartj/ehz386 31212315

[25] Liao J‐N , Chao T‐F , Liu C‐J , et al. Risk and prediction of dementia in patients with atrial fibrillation ‐ a nationwide population‐based cohort study. Int J Cardiol. 2015;199:25‐30. 10.1016/j.ijcard.2015.06.170 26173170

[26] Marengoni A , Qiu C , Winblad B , Fratiglioni L . Atrial fibrillation, stroke and dementia in the very old: a population‐based study. Neurobiol Aging. 2011;32(7):1336‐1337. 10.1016/j.neurobiolaging.2009.08.002 19732992

[27] Marzona I , Baviera M , Vannini T , et al. Risk of dementia and death in patients with atrial fibrillation: a competing risk analysis of a population‐based cohort. Int J Cardiol. 2016;220:440‐444. 10.1016/j.ijcard.2016.06.235 27394970

[28] Marzona I , O’Donnell M , Teo K , et al. Increased risk of cognitive and functional decline in patients with atrial fibrillation: results of the ONTARGET and TRANSCEND studies. CMAJ (Can Med Assoc J). 2012;184(6):E329‐E336. 10.1503/cmaj.111173 22371515 PMC3314061

[29] Nah M‐A , Lee KS , Hwang T‐Y . Association between atrial fibrillation and the risk of dementia in the Korean elderly: a 10‐year nationwide cohort study. J Prev Med Public Health. 2020;53(1):56‐63. 10.3961/jpmph.19.117 32023675 PMC7002991

[30] Rastas S , Verkkoniemi A , Polvikoski T , et al. Atrial fibrillation, stroke, and cognition. Stroke. 2007;38(5):1454‐1460. 10.1161/STROKEAHA.106.477299 17395865

[31] Singh‐Manoux A , Fayosse A , Sabia S , et al. Atrial fibrillation as a risk factor for cognitive decline and dementia. Eur Heart J. 2017;38(34):2612‐2618. 10.1093/eurheartj/ehx208 28460139 PMC5837240

[32] Haring B , Leng X , Robinson J , et al. Cardiovascular disease and cognitive decline in postmenopausal women: results from the women's health initiative memory study. J Am Heart Assoc. 2013;2(6):e000369. 10.1161/JAHA.113.000369 24351701 PMC3886762

[33] Alonso A , Arenas de Larriva AP . Atrial fibrillation, cognitive decline and dementia. Eur Cardiol Rev. 2016;11(1):49. 10.15420/ecr.2016:13:2 PMC4988519 27547248

[34] Kim M‐S , Kim J‐J . Heart and brain interconnection ‐ clinical implications of changes in brain function during heart failure. Circ J. 2015;79(5):942‐947. 10.1253/circj.CJ-15-0360 25891994

[35] De La Torre JC . Is Alzheimer's disease a neurodegenerative or a vascular disorder? Data, dogma, and dialectics. Lancet Neurology. 2004;3(3):184‐190. 10.1016/S1474-4422(04)00683-0 14980533

[36] Zuliani G , Trentini A , Rosta V , et al. Increased blood BACE1 activity as a potential common pathogenic factor of vascular dementia and late onset Alzheimer's disease. Sci Rep. 2020;10(1):14980. 10.1038/s41598-020-72168-3 32917964 PMC7486910

[37] Allessie M , Ausma J , Schotten U . Electrical, contractile and structural remodeling during atrial fibrillation. Cardiovasc Res. 2002;54(2):230‐246. 10.1016/s0008-6363(02)00258-4 12062329

[38] Nattel S . New ideas about atrial fibrillation 50 years on. Nature. 2002;415(6868):219‐226. 10.1038/415219a 11805846

[39] Wijffels MCEF , Kirchhof CJHJ , Dorland R , Allessie MA . Atrial fibrillation begets atrial fibrillation. Circulation. 1995;92(7):1954‐1968. 10.1161/01.CIR.92.7.1954 7671380

[40] Divo MJ , Martinez CH , Mannino DM . Ageing and the epidemiology of multimorbidity. Eur Respir J. 2014;44(4):1055‐1068. 10.1183/09031936.00059814 25142482 PMC4918092

[41] Piccirillo JF , Vlahiotis A , Barrett LB , Flood KL , Spitznagel EL , Steyerberg EW . The changing prevalence of comorbidity across the age spectrum. Crit Rev Oncology/Hematology. 2008;67(2):124‐132. 10.1016/j.critrevonc.2008.01.013 PMC2536650 18375141

[42] Hsu JY , Liu PPS , Liu AB , Lin SM , Huang HK , Loh CH . Lower risk of dementia in patients with atrial fibrillation taking non‐vitamin K antagonist oral anticoagulants: a nationwide population‐based cohort study. J Am Heart Assoc. 2021;10(5):e016437. 10.1161/JAHA.120.016437 33586465 PMC8174264

[43] Bezabhe WM , Bereznicki LR , Radford J , et al. Factors influencing oral anticoagulant use in patients newly diagnosed with atrial fibrillation. Eur J Clin Invest. 2021;51(5):e13457. 10.1111/eci.13457 33222261

[44] Viscogliosi G , Ettorre E , Chiriac IM . Dementia correlates with anticoagulation underuse in older patients with atrial fibrillation. Archives Gerontology Geriatrics. 2017;72:108‐112. 10.1016/j.archger.2017.05.014 28622606

[45] Kim D , Yang P‐S , Joung B . Prevention of dementia in patients with atrial fibrillation. Korean Circ J. 2021;51(4):308‐319. 10.4070/kcj.2021.0027 33821580 PMC8022029

[46] Gaita F , Corsinovi L , Anselmino M , et al. Prevalence of silent cerebral ischemia in paroxysmal and persistent atrial fibrillation and correlation with cognitive function. J Am Coll Cardiol. 2013;62(21):1990‐1997. 10.1016/j.jacc.2013.05.074 23850917

[47] Dagres N , Chao T‐F , Fenelon G , et al. European heart rhythm association (EHRA)/Heart rhythm society (HRS)/Asia pacific heart rhythm society (APHRS)/Latin American heart rhythm society (LAHRS) expert consensus on arrhythmias and cognitive function: what is the best practice? Europace. 2018;20(9):1399‐1421. 10.1093/europace/euy046 29562326 PMC6658813

[48] Dietzel J , Haeusler KG , Endres M . Does atrial fibrillation cause cognitive decline and dementia? EPP Eur. 2018;20(3):408‐419. 10.1093/europace/eux031 28387847

[49] Ng KH , Hart RG , Eikelboom JW . Anticoagulation in patients aged ≥75 years with atrial fibrillation: role of novel oral anticoagulants. Cardiol Ther. 2013;2(2):135‐149. 10.1007/s40119-013-0019-y 25135392 PMC4107426

[50] Anselmino M , Scarsoglio S , Saglietto A , Gaita F , Ridolfi L . Transient cerebral hypoperfusion and hypertensive events during atrial fibrillation: a plausible mechanism for cognitive impairment. Sci Rep. 2016;6(1):28635. 10.1038/srep28635 27334559 PMC4917883

[51] Bunch TJ , Crandall BG , Weiss JP , et al. Patients treated with catheter ablation for atrial fibrillation have long‐term rates of death, stroke, and dementia similar to patients without atrial fibrillation. J Cardiovasc Electrophysiol. 2011;22(8):839‐845. 10.1111/j.1540-8167.2011.02035.x 21410581

[52] Islam MM , Poly TN , Walther BA , et al. Association between atrial fibrillation and dementia: a meta‐analysis. Front Aging Neurosci. 2019;11:305. 10.3389/fnagi.2019.00305 PMC6857071 31780919

[53] Santangeli P , Di Biase L , Bai R , et al. Atrial fibrillation and the risk of incident dementia: a meta‐analysis. Heart Rhythm. 2012;9(11):1761‐1768. 10.1016/j.hrthm.2012.07.026 22863685

