# Supplementary figures and images for: Risk of dementia in patients with atrial fibrillation: Short versus long follow‐up. A systematic review and meta‐analysis
Source: Int J Geriatr Psychiatry. 2021 May 27;36(10):1488–500. doi: 10.1002/gps.5582 (PMC8518611; doi:10.1002/gps.5582)

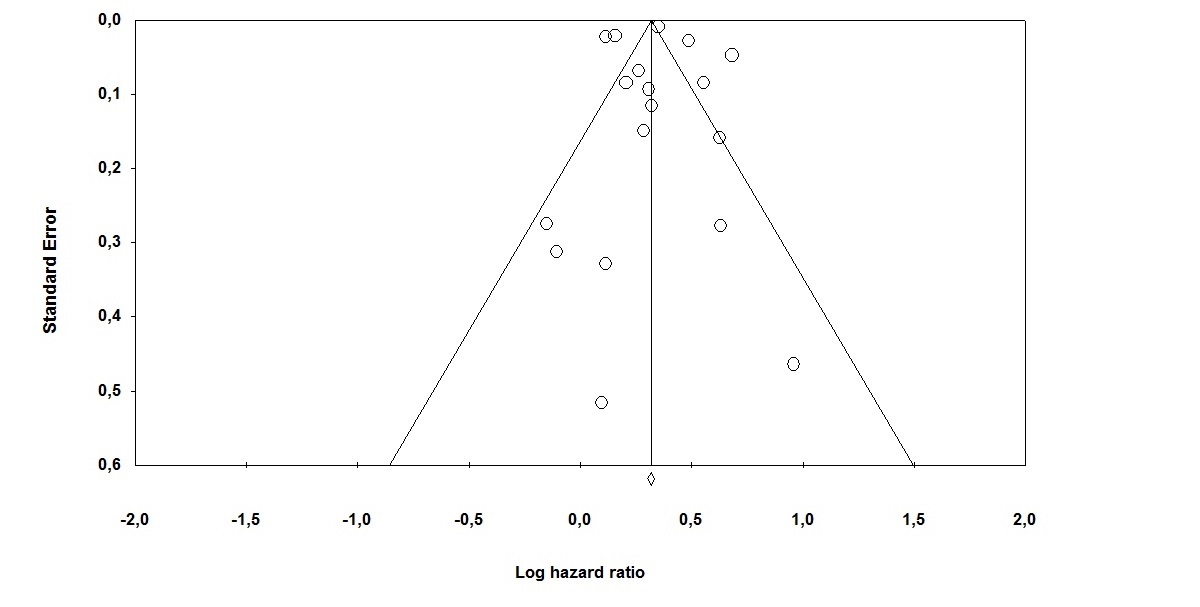

Supplement: Supplementary file 1 — Supplementary Material 1 [file GPS-36-1488-s005.jpg]

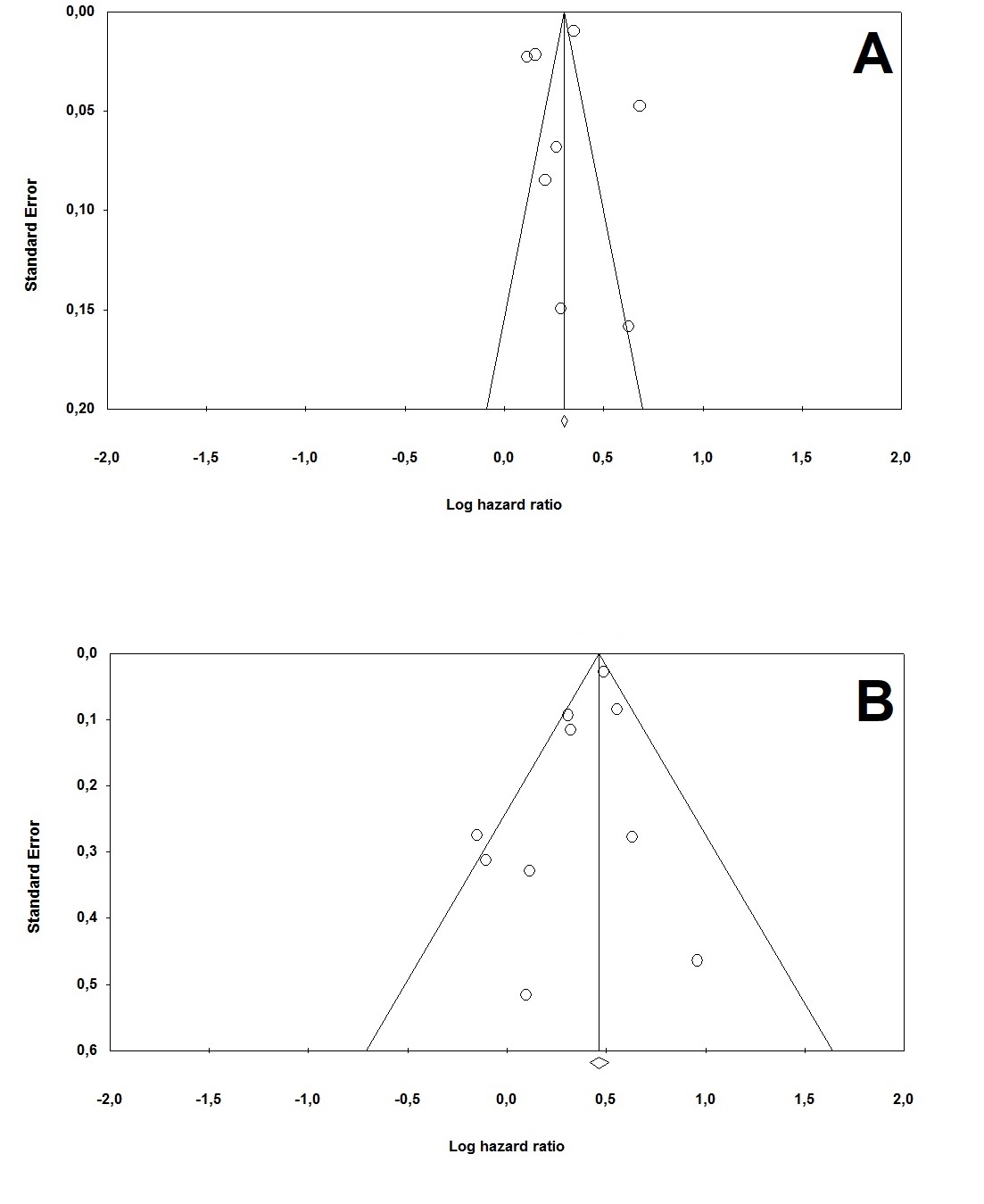

Supplement: Supplementary file 2 — Supplementary Material 2 [file GPS-36-1488-s004.jpg]

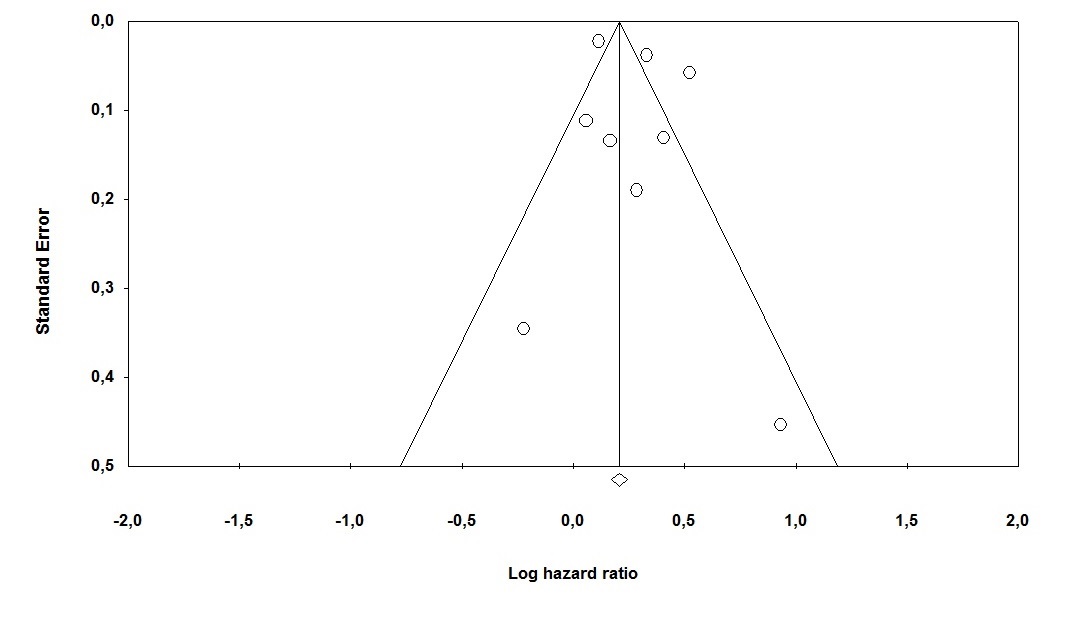

Supplement: Supplementary file 3 — Supplementary Material 3 [file GPS-36-1488-s003.jpg]
