# Supplementary material for: Risk of dementia in patients with atrial fibrillation: Short versus long follow‐up. A systematic review and meta‐analysis
Source: Int J Geriatr Psychiatry. 2021 May 27;36(10):1488–500. doi: 10.1002/gps.5582 (PMC8518611; doi:10.1002/gps.5582)
Supplement: Supplementary file 5 — Supplementary Material 5 [file GPS-36-1488-s002.docx]

**Supplementary table 1**. Additional general characteristics of the studies included into the meta-analysis.

| Author | Year | Univariate HR | comorbidities | Use of oral anticoaugulant drug | Previous/subsequent Stroke or TIA | Alzheimer’s disease outcome  (adjusted HR) |
| --- | --- | --- | --- | --- | --- | --- |
| Kim et al. | 2020 | 1.28 (1.23-1.33) | Diabetes: 7.7%  Heart failure: 2.6% | 0.05% | 0/NR* | 1.69 (1.51-1.90) |
| Nah et al. | 2020 | NR | Diabetes: 20.9%  Heart failure: 3.1%  Ischemic heart disease:23.8% Valvular heart disease: 0.7% | NR | 0/NR | 1.12 (1.07, 1.17) |
| Kim et al. | 2019 | NR | Diabetes: 14.3%  Heart failure: 6.3% | 0.12% | 0/NR* | 1.39 (1.29-1.50) |
| Krawczyk et al. | 2019 | NR | Diabetes: 24.3 %  Congestive heart failure: 7.4%  Coronary heart disease:24.8%  Myocardial infraction: 13.5% | 15.3% | Only patients with first-ever ischaemic stroke were included | NR |
| Ding et al. | 2018 | NR | Diabetes: 9.4 %  Heart failure: 9.4%  Coronary heart disease:13.9% | NR | 2.6%/4.3% | 1.33 (0.92-1.94) |
| Chen et al. | 2018 | NR | Diabetes: 15 %  Heart failure: 4%  Coronary heart disease:5% | 8% | 2%/NR | NR |
| Singh-Manoux et al. | 2017 | NR | NR* | NR | 0/NR | NR |
| Marzona et al. | 2016 | 1.72 (1.64-1.79) | NR* | NR* | NR* | NR |
| De Bruijn et al. | 2015 | NR | Diabetes: 10.3 %  Heart failure: 8.0%  Coronary heart disease:2.8% | 22.6% | 0/5.7% | 1.18 (0.91-1.54) |
| Liao et al. | 2015 | 1.426(1.403-1.450) | Diabetes: 27.8 %  Vascular disease: 8.8% | 14.2% | NR/NR | NR |
| Rusanen et al. | 2014 | NR | Heart disease (Myocardial infraction, Coronary heart disease and angina pectoris): 33.9%  Diabetes: 10.7% | NR | 10.7%/NR | 2.54 (1.04-6.16) |
| Haring et al. | 2013 | NR | Myocardial infraction: 4.0%^#^  Angina pectoris: 9.0%  Congestive heart failure: 1.3%  Diabetes: 6.1% | 7.5% | 0/5.4% | NR |
| Marzona et al. | 2012 | NR | Diabetes: 37.2%  Myocardial infraction: 48.5% | NR^§^ | 21%/4.3% | NR |
| Dublin et al. | 2011 | NR | Coronary heart disease:18.8%  Congestive heart failure: 3.4%  Diabetes: 9.4% | 4.5% | 0/NR | 1.50 (1.16-1.94) |
| Bunch et al. | 2010 | NR | NR* | NR | NR | NR |
| Marengoni et al. | 2011 | NR | NR | NR | 0/1.9% | 0.8 (0.4-1.5) |
| Rastas et al. | 2007 | NR | Diabetes: 20.2%  Myocardial infraction: 14.1%  Heart failure: 60.1% | 1.9% | 20.1%/NR | NR |
| Forti et al. | 2006 | NR | NR | NR | NR/NR | NR* |

NR, Not reported

^*^it is only reported the percentage of stroke or comorbidities in separate groups (e.g. AF and no AF ) but not in the total sample

^§^ It is only reported the total percentage of subjects taking anti-coagulant therapy alone or in combination with antiplatelet therapy

#the percentage was calculated in subjects with no incident stroke
